# Supplementary material for: Particle velocity controls phase transitions in contagion dynamics
Source: Sci Rep. 2019 Apr 23;9:6463. doi: 10.1038/s41598-019-42871-x (PMC6478726; doi:10.1038/s41598-019-42871-x)
Supplement: Supplementary file 1 — Supplementary Information [file 41598_2019_42871_MOESM1_ESM.pdf]

Particle velocity controls phase transitions in contagion dynamics

SUPPLEMENTARY INFORMATION

Jorge P. Rodríguez, Fakhteh Ghanbarnejad, and Víctor M. Eguíluz

LIST OF SUPPLEMENTARY FIGURES

I Temporal contact network

Supplementary Figure S1

II Single infection in an uncorrelated contact sequence

Supplementary Figure S2

III Single infection: $\mathbf{v = 0}$ and $\mathbf{v = 0.5d}$

Supplementary Figures S3 and S4

IV Cooperative contagion with static particles

Supplementary Figures S5, S6, S7, S8 and S9

V Cooperative contagion in an uncorrelated contact sequence

Supplementary Figures S10, S11 and S12

I. TEMPORAL CONTACT NETWORK

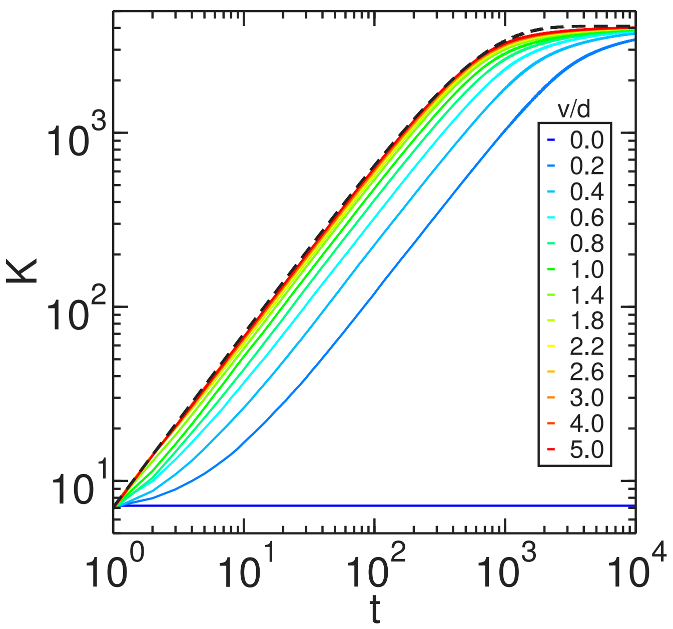

Supplementary Figure S1. Time evolution of the number of accumulated contacts K of an average particle (*i.e.*, number of particles with which it had at least one contact), for $d = 30$ and different velocities, from $v = 0.0$ (blue) to $v = 5.0d$ (red). The black dashed line represents the uncorrelated contact network limit ($v \to \infty$), given by $K(t) = (N-1)(1 - e^{-\pi d^2 t/L^2})$.

II. SINGLE INFECTION IN AN UNCORRELATED CONTACT SEQUENCE

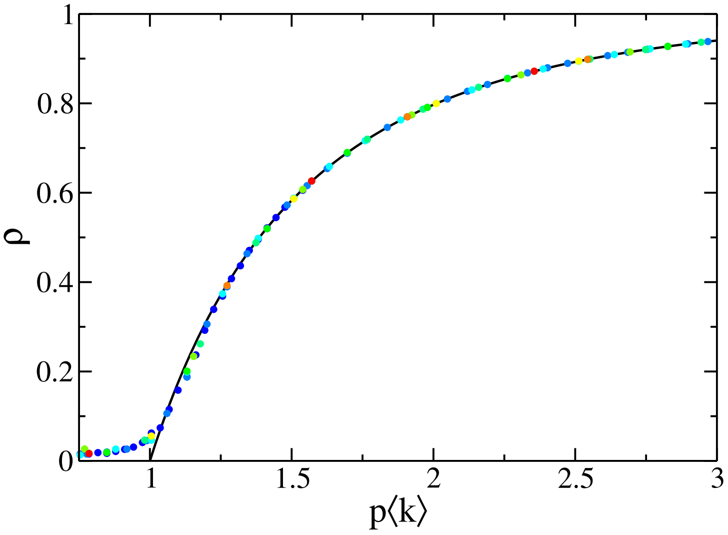

Supplementary Figure S2. Universal behavior of the density of recovered particles ρ in the final absorbing configuration, for different interaction radii d (from $d = 20$ (blue) to $d = 100$ (red), with colors varying continuously between these limits with $\Delta d = 10$), when the control parameter is rescaled to $p\langle k\rangle$. The solid line represents the mean-field solution, given by $\rho = 1 - \mathrm{e}^{p\langle k\rangle\rho}$.

III. SINGLE INFECTION: v = 0 AND v = 0.5d

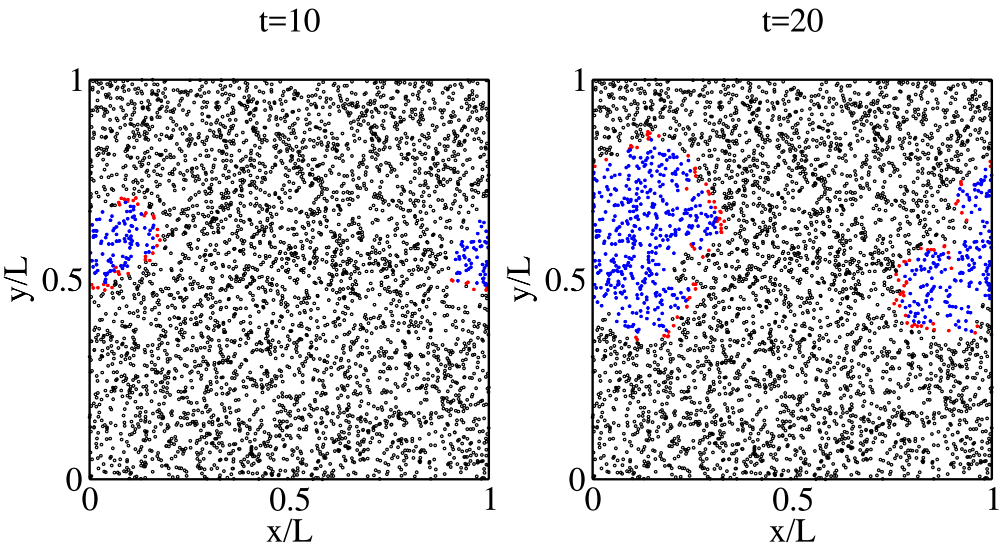

Supplementary Figure S3. Visualization of the contagion process at $t = 10$ and $t = 20$ with $p = 1$, $d = 30$ and $v = 0$. Recovered particles (blue symbols) are connected only to whether other recovered or infected particles (red symbols), and not with susceptible particles (empty symbols).

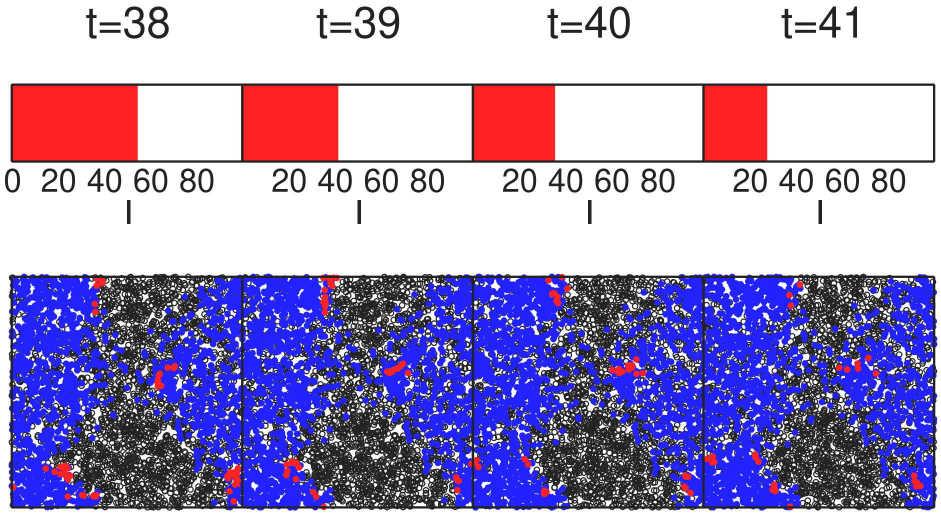

Supplementary Figure S4. Visualization of the contagion process at $t = 38$, $t = 39$, $t = 40$ and $t = 41$ with $p = 1$, $d = 28$ and $v = 0.5d$. Infected particles (red symbols) are more likely connected to recovered particles (blue symbols), than to susceptible particles (empty symbols). The red upper bars represent the number of infected particles I.

IV. COOPERATIVE CONTAGION WITH STATIC PARTICLES

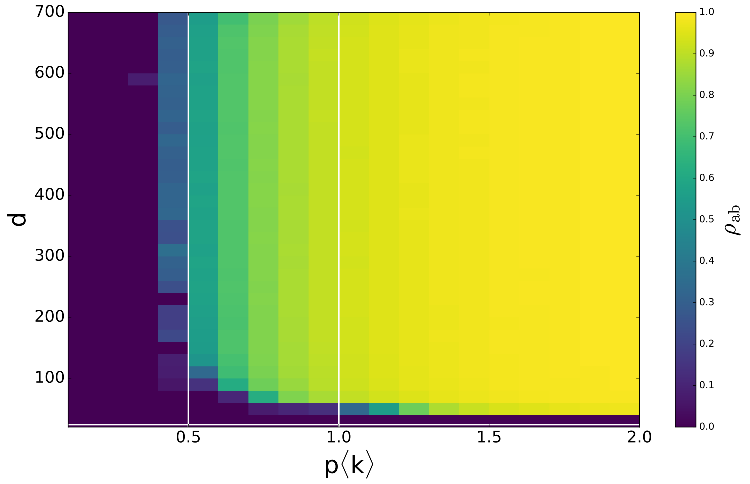

Supplementary Figure S5. Density of doubly recovered particles ρ_{ab} in the final absorbing configuration as a function of the normalized primary infection probability $p\langle k\rangle$ and the interaction radius d, for $q = 1$. The white horizontal line represents $d = d_c$ and the vertical lines indicate the cases $p\langle k\rangle = 0.5$ and $p\langle k\rangle = 1$.

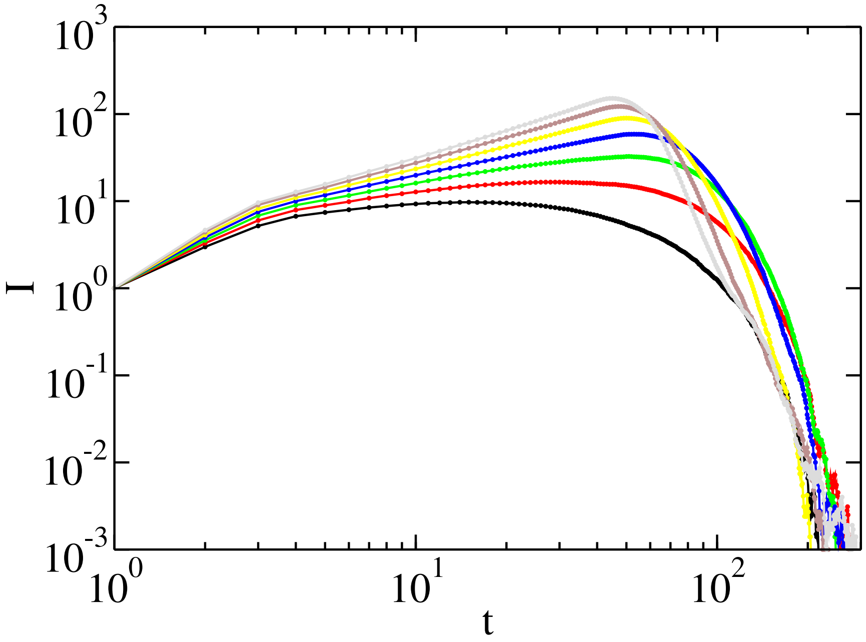

Supplementary Figure S6. Time evolution of the number of infected particles I for a system of static particles with $d = 30$ and $q = 1$. The plotted values of $p\langle k \rangle$ are, from the black to the grey curve, of 1.7, 1.9, 2.1, 2.3, 2.5, 2.7 and 2.9.

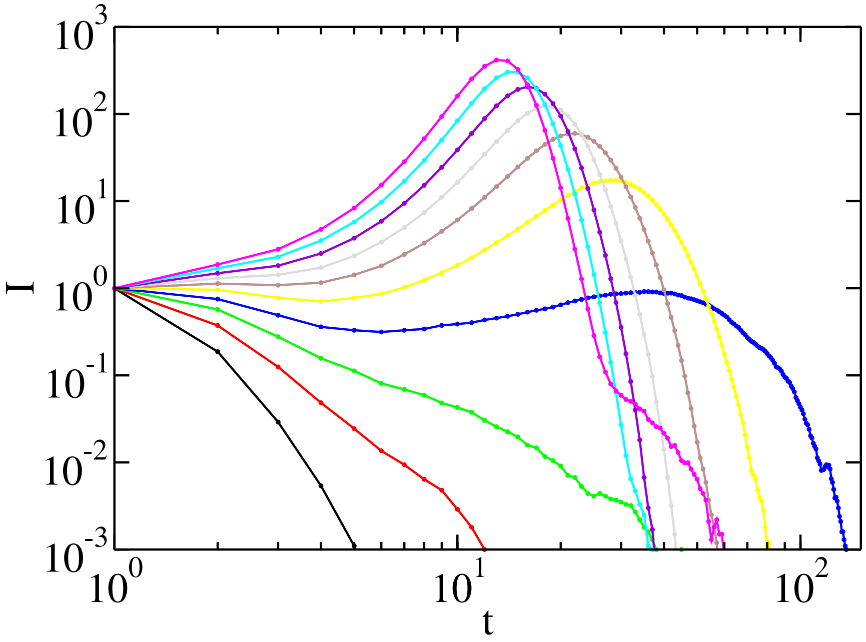

Supplementary Figure S7. Time evolution of the number of infected particles I for a system of static particles with $d = 700$ and $q = 1$. The plotted values of $p\langle k\rangle$ are, from the black to the magenta curve, of 0.5, 0.6, 0.7, 0.8, 0.9, 1.0, 1.1, 1.2, 1.3 and 1.4.

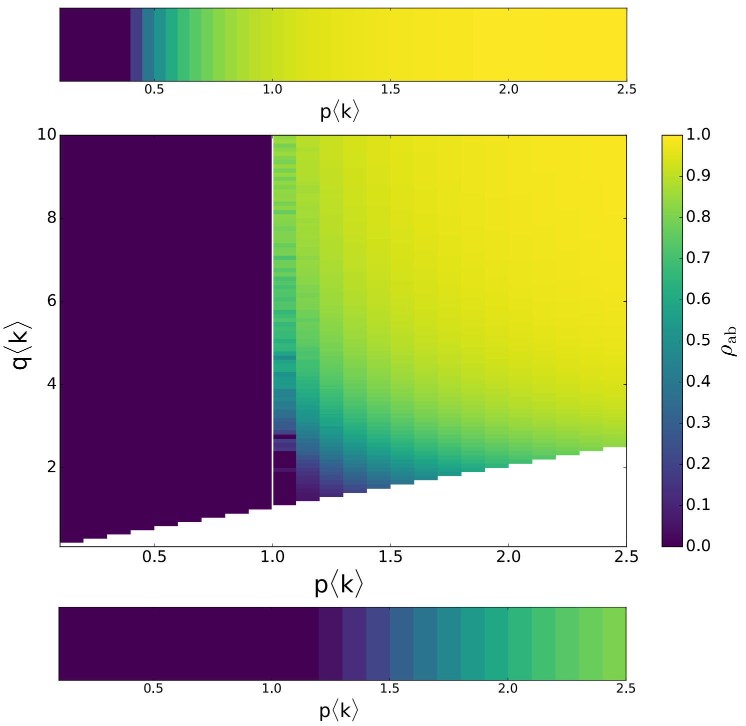

Supplementary Figure S8. Density of doubly recovered particles in the final absorbing configuration, as a function of p and q, for $d = 700 \gg d_c$. The upper and lower plots represent, respectively, the limit cases $q = 1$ and $q = p$, and the white line in the central plot indicates the limit $p\langle k\rangle = 1$.

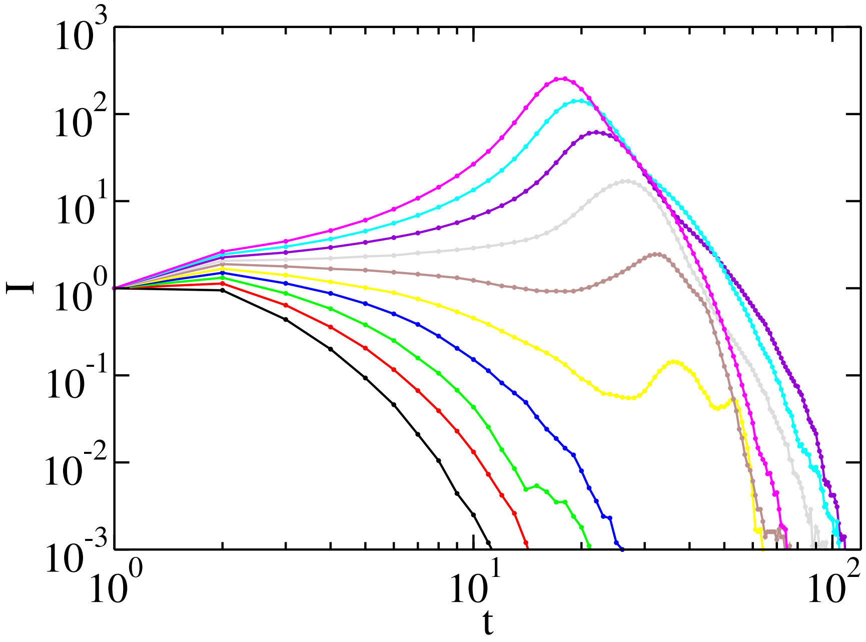

Supplementary Figure S9. Time evolution of the number of infected particles for a system of static particles with $d = 700$ and $q\langle k\rangle = 10$. The plotted values of $p\langle k\rangle$ are, from the black to the magenta curve, of 0.1, 0.2, 0.3, 0.4, 0.5, 0.6, 0.7, 0.8, 0.9 and 1.0.

V. COOPERATIVE CONTAGION IN AN UNCORRELATED CONTACT SEQUENCE

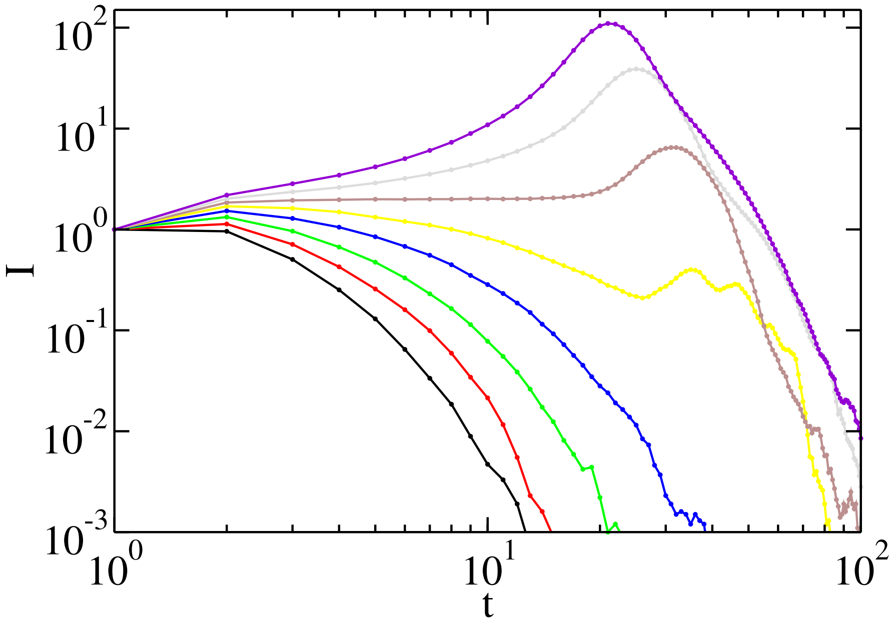

Supplementary Figure S10. Time evolution of the number of infected particles for $d = 30$, infinite velocity and $q\langle k\rangle = 3$. The plotted values of $p\langle k\rangle$ are, from the black to the violet curve, of 0.5, 0.6, 0.7, 0.8, 0.9, 1.0, 1.1, and 1.2.

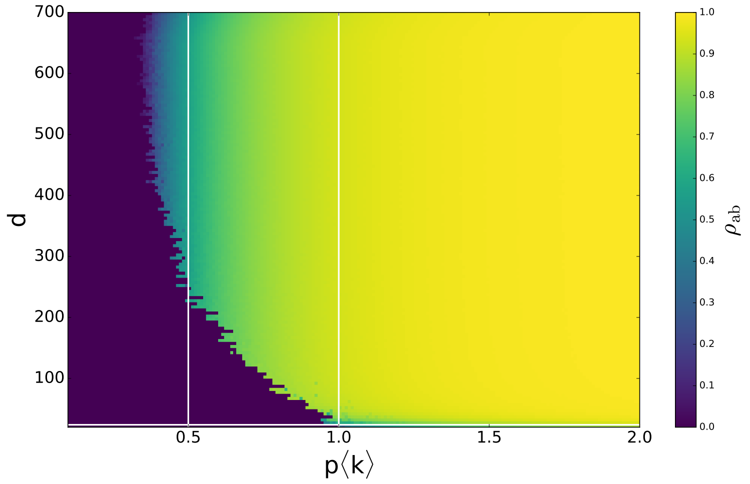

Supplementary Figure S11. Cooperative contagion on systems of particles with infinite velocity and $q = 1$. As d is increased ($d_c < d \ll d_{\max}$), a continuous phase transition becomes discontinuous, coming back to a continuous phase transition for high interaction ranges, $d \sim L$. The white horizontal line represents $d = d_c$ and the vertical lines indicate the cases $p\langle k\rangle = 0.5$ and $p\langle k\rangle = 1$.

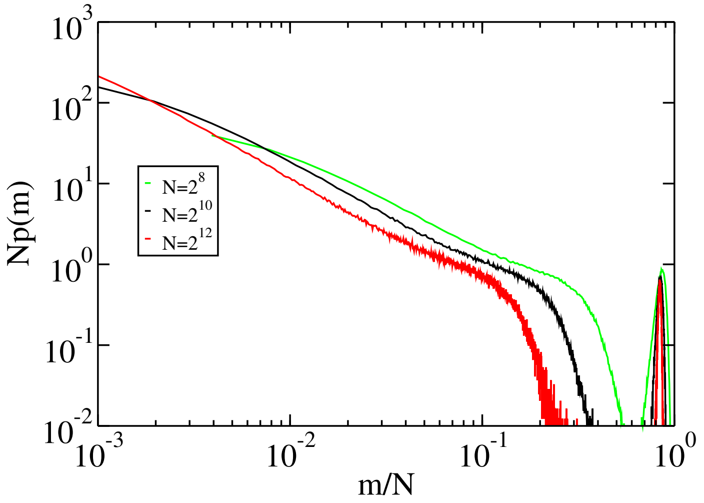

Supplementary Figure S12. Distribution of the number of recovered particles $m = 1-S$ in the final absorbing configuration, for $d = 30$, infinite velocity, $p\langle k\rangle = 1$, and $q = 1$, and different system sizes N, keeping the density $\sigma = N/L^2$ constant. There is a gap between the low prevalence configuration, leading to a fraction of recovered nodes which decays as a power-law, and the high prevalence configuration, which leads to a fraction of recovered nodes which scales linearly with the system size.
